# The influence of sarcopenia in dropped head syndrome in older women

**DOI:** 10.1186/s13013-017-0110-6

**Published:** 2017-02-22

**Authors:** Yawara Eguchi, Toru Toyoguchi, Masao Koda, Munetaka Suzuki, Hajime Yamanaka, Hiroshi Tamai, Tatsuya Kobayashi, Sumihisa Orita, Kazuyo Yamauchi, Miyako Suzuki, Kazuhide Inage, Kazuki Fujimoto, Hirohito Kanamoto, Koki Abe, Yasuchika Aoki, Kazuhisa Takahashi, Seiji Ohtori

**Affiliations:** 1 Department of Orthopeadic Surgery, Shimoshizu National Hospital, 934-5, Shikawatashi, Yotsukaido, Chiba 284-0003 Japan; 2 Department of Orthopaedic Surgery, Chiba Qiball Clinic, 4-5-1, Chuo-ku, Chiba, 260-0013 Japan; 3 Department of Orthopaedic Surgery, Graduate School of Medicine, Chiba University, 1-8-1 Inohana, Chuo-ku, Chiba, 260-8670 Japan; 4 Department of Orthopaedic Surgery, Eastern Chiba Medical Center, 3-6-2, Okayamadai, Togane, Chiba 283-8686 Japan

**Keywords:** Dropped head syndrome, Sarcopenia, Skeletal muscle, Bioelectrical impedance analyzer

## Abstract

**Background:**

Age-related sarcopenia may cause physical dysfunction. We investigated the involvement of sarcopenia in dropped head syndrome (DHS).

**Methods:**

Our study subjects were ten elderly women with idiopathic DHS (mean age 75.1 years, range 55–89). Twenty age- and sex-matched volunteers (mean age 73.0, range 58–83) served as controls. We used a bioelectrical impedance analyzer (BIA) to analyze body composition, including appendicular skeletal muscle mass index (SMI; appendicular lean mass (kg)/(height (m))^2^). SMI <5.75 was considered diagnostic for sarcopenia. Cervical sagittal plane alignment: C2–7 sagittal vertical axis (SVA), C2–7 angle (C2–C7 A), and C2 slope (C2S) were also measured. We investigated sarcopenia prevalence in both groups, height, weight, BMI, lean mass arm, lean mass leg, lean mass trunk, appendicular lean mass, total lean mass, and SMI. In addition, we also examined the correlation between cervical spine alignment and SMI in DHS.

**Results:**

Sarcopenia was observed at a high rate in DHS subjects: 70% compared to 25% of healthy controls. Height, weight, BMI, lean mass arm, lean mass leg, axial lean mass, appendicular lean mass, total lean mass, and SMI all had significantly lower values in the DHS group. In particular, total lean mass, lean mass arm, and lean mass trunk were considerably lower in the DHS group. There was no correlation noted between cervical spine alignment and SMI.

**Conclusions:**

Sarcopenia prevalence was high in the DHS group—70 versus 25% in the control group, suggesting the involvement of sarcopenia in DHS. In particular, axial lean mass and lean mass arm were markedly reduced in the DHS group. DHS is due to significant weakness of the neck extensor group, and chin-on-chest deformity occurs. Until the present, evaluation of DHS has been done using only MRI; no studies have systematically examined skeletal muscle mass. In the present study, muscle mass decrease was noted not only in the neck muscles but also throughout the entire body. Involvement of trunk and upper limb muscles in particular suggests a disuse atrophy of the upper body and spinal muscles. BIA can easily and systemically evaluate skeletal muscle mass. We expect it to contribute to further elucidating the pathogenesis of DHS.

## Background

Dropped head syndrome (DHS) exhibits chin-on-chest deformity due to significant weakness of the neck extensor group [1–6]. DHS can impair quality of life, resulting in restrictions on forward gaze and ambulation, dysphagia, and neck pain. Neck extensor atrophy occurs in a variety of disease backgrounds, including neurological, neuromuscular, and muscular disorders [1, 2]. Among these, idiopathic DHS, due to neck extensor muscle failure of unknown cause, is a problem for many elderly patients. There is the possibility for further increase as society ages [5].

Sarcopenia is a syndrome characterized by progressive and systemic reduction in skeletal muscle mass. It carries a high risk of becoming bedridden from a fall, and there is great physical and economic loss in an aging society [7–10]. It is believed that sarcopenia results from inactivity, but the mechanism is not entirely clear. Decrease in back strength due to sarcopenia is believed to contribute to the development of DHS. Until the present, evaluation of DHS has consisted of only local neck MRI 5,6. There has been no report of the involvement of whole-body skeletal muscle mass in sarcopenia.

In the present study, we report the prevalence of sarcopenia in DHS and examine whole-body skeletal muscle mass.

## Methods

Our study subjects were ten elderly women with idiopathic DHS (mean age 75.1 years, range 55–89). Twenty age- and sex-matched volunteers (mean age 73.0, range 58–83) served as controls. DHS was clinically defined as a disabling condition in which severe weakness of the neck extensor muscles causes difficulty in lifting the head against gravity, which results in a correctable chin-on-chest deformity. Two patients with a single thoracolumbar compression fracture (case 1: L3, case 3: Th12) were included (Table 1). Subjects were excluded for multiple thoracolumbar compression fractures or a history of spinal surgery.

**Table 1:** Patient characteristics

						Skeletal muscle						Cervical sagittal alignment		
Case	Age	Sex	Height (m)	Weight (kg)	BMI (kg/m2)	Lean mass arm (kg)	Lean mass leg (kg)	Lean mass trunk (kg)	Appendicular lean mass (kg)	Total lean mass (kg)	SMI (kg/m2)	C2–7 SVA (m)	C2–7 A (°)	C2S (°)
1	73	F	1.47	51.3	23.74	2.93	9.34	14.2	12.27	26.47	5.678 ^a^	30	−22	36
2	69	F	1.47	35.3	16.33	1.6	7.16	10.6	8.76	19.36	4.053 ^a^	35	16	17
3	82	F	1.4	40.8	20.81	2.26	7.81	11.6	10.07	21.67	5.137 ^a^	63	−13	55
4	77	F	1.44	33.4	16.10	1.74	8.1	10.6	9.84	20.44	4.745 ^a^	52	−14	44
5	55	F	1.4	49.9	25.45	2.34	10.32	12.1	12.66	24.76	6.459	64	−59	90
6	60	F	1.54	45.2	19.05	2.55	9.23	13.6	11.78	25.38	4.967 ^a^	61	−56	50
7	89	F	1.47	48.3	22.35	2.58	12.29	12.7	14.87	27.57	6.881	60	20	30
8	88	F	1.43	43.5	21.27	2.29	10.1	11.9	12.39	24.29	6.058	47	−14	35
9	74	F	1.4	39.7	20.25	2.83	8.01	13.1	10.84	23.94	5.530 ^a^	69	−50	80
10	84	F	1.46	52.9	24.81	2.83	8.77	14	11.6	25.6	5.441 ^a^	30	−1	28
Mean	75.1		1.448	44.03	21.02	2.395	9.113	12.44	11.508	23.948	5.495	51.1	−19.3	46.5

^a^Sarcopenia positive (SMI <5.75)

A multi-frequency bioelectrical impedance analyzer (BIA), the InBody 720 Biospace device (Biospace Co., Ltd., Korea), was used according to the manufacturer’s guidelines. BIA estimates body composition using the difference of conductivity of the various tissues due to the difference of their biological characteristics. Conductivity is proportional to water content (more specifically to electrolytes), and conductivity decreases as the cell approaches a perfect spherical shape. Adipose tissue is composed of round cells and contains relatively little water compared to other tissues like muscle; therefore, conductivity decreases as body fat increases. In practice, electrodes are placed at eight precise tactile points of the body to achieve a multi-segmental frequency analysis. A total of 30 impedance measurements are obtained using six different frequencies (1, 5, 50, 250, 500, and 1000 kHz) for the following five segments of the body: right and left arms, trunk, and right and left legs.

Appendicular skeletal muscle mass was calculated as the sum of skeletal muscle mass in the arms and legs, assuming that mass of lean soft tissue is effectively equivalent to skeletal muscle mass. Appendicular skeletal mass index (SMI) was determined as the sum of arm and leg lean mass (kg)/(height (m))^2^. The diagnosis of sarcopenia among women was defined as appendicular SMI value <5.75 kg/m^2^, determined using sarcopenia normative data [11].

**Fig. 1:**
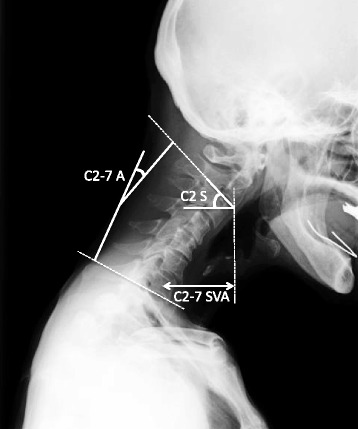
Cervical sagittal plane alignment. C2–7 sagittal vertical axis (C2–7 SVA), C2–7 angle (C2–C7 A), and C2 slope (C2 S) were measured. C2–7 A was minus in the kyphotic direction and positive in the lordotic direction

The radiographs were taken in the standing position. Cervical sagittal plane alignment was measured by C2–7 sagittal vertical axis (SVA), C2–7 angle (C2–C7 A), and C2 slope (C2 S) (Fig. 1).

We investigated sarcopenia prevalence in both groups, height, weight, BMI, lean mass arm, lean mass leg, lean mass trunk, appendicular lean mass, total lean mass, and SMI (Table 1). In addition, we also examined the correlation between cervical spine alignment and SMI in DHS.

### Statistical analysis

Statistical analyses were performed with StatView software (version 5.0).

For each parameter, differences between groups were evaluated using unpaired *t* test.

Pearson correlation coefficients were calculated to determine the correlation between appendicular SMI and spinal parameters. A threshold of *p* < 0.05 was considered significant.

## Results

**Fig. 2:**
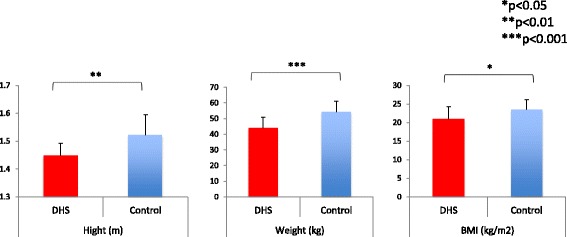
Height, weight, and BMI results. Height, weight, and BMI were significantly lower in the DHS group

Height, weight, and BMI were significantly lower for the DHS group compared to controls (Fig. 2): height for the DHS group was 1.448 ± 0.044 m compared to 1.522 ± 0.072 m for controls (*p* = 0.0072); weight for the DHS group was 44.03 ± 6.70 kg compared to 54.33 ± 6.65 kg for controls (*p* = 0.0005); and BMI for the DHS group was 21.02 ± 3.23 kg/m^2^ compared to 23.48 ± 2.65 kg/m^2^ for controls (*p* = 0.036).

Cervical spine parameters for the DHS group were C2–7SVA: 51.1 ± 14.8 mm, C2–7A: −19.3 ± 28.1°, and C2S: 46.5 ± 23.2°, representing advanced anteversion and kyphosis. We detected no correlation of any of these parameters with SMI: C2–7SVA (*r* = 0.300, *p* = 0.39), C2–7A (*r* = −0100, *p* = 0.78), and C2S (*r* = 0.301, *p* = 0.39).

**Fig. 3:**
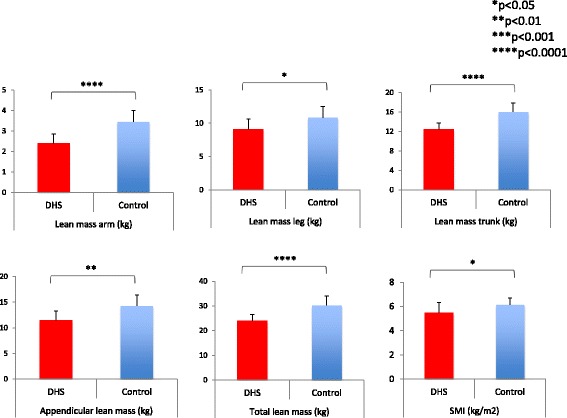
Skeletal muscle mass results. Lean mass arm was 2.39 kg for the DHS group and 3.45 kg for controls (*p* < 0.0001), lean mass leg was 9.11 kg for DHS and 10.86 kg for controls (*p* < 0.05), lean mass trunk was 12.44 kg for DHS and 16.02 kg for controls (*p* < 0.0001), appendicular lean mass was 11.50 kg for DHS and 14.31 kg for controls (*p* < 0.01), total lean mass was 23.94 kg for DHS and 30.33 kg for controls (*p* < 0.0001), and SMI was 5.49 kg/m^2^ for DHS and 6.15 kg/m^2^ for controls (*p* < 0.05). All items were significantly lower in the DHS group

Sarcopenia prevalence was high in the DHS group, including 7 out of 10 cases (70%), versus 5 out of 20 controls (20%). Regarding skeletal muscle mass parameters: lean mass arm was 2.39 ± 0.44 kg in the DHS group versus 3.45 ± 0.54 kg in controls (*p* = 0.000016), lean mass leg was 9.11 ± 1.50 kg in DHS versus 10.86 ± 1.67 kg in controls (*p* = 0.010), lean mass trunk was 12.44 ± 1.30 kg in DHS versus 16.02 ± 1.81 kg in controls (*p* = 0.0000081), appendicular lean mass was 11.50 ± 1.72 kg in DHS versus 14.31 ± 2.06 kg in controls (*p* = 0.0011), total lean mass was 23.94 ± 2.65 kg in DHS versus 30.33 ± 3.74 kg in controls (*p* = 0.000062), and SMI was 5.49 ± 0.83 kg/m^2^ in DHS versus 6.15 ± 0.54 kg/m^2^ in controls (*p* = 0.016). The DHS group had significantly lower values for all items (Fig. 3).

## Discussion

Dropped head syndrome comprises a group of disorders associated with chin-on-chest deformity due to marked weakness of the neck extensor muscles. It has been reported in the setting of a wide variety of diseases, including neurological, neuromuscular, and muscular disorders [1–6], Parkinson disease [3], multiple system atrophy [4], amyotrophic lateral sclerosis, and isolated neck extensor myopathy (INEM). Katz et al. reported on DHS due to cervical extensor muscle weakness of unknown origin in INEM. They propose an isolated myopathy that occurs due to nonspecific inflammation in the extensor muscle due to continued abnormal posture. This is observed in those greater than 60 years of age and mainly confined to the neck extensor muscles. A subacute process is recognized that includes weakness of shoulder blade and upper arm muscles, myogenic changes in needle EMG, and muscle atrophy in MRI, but these features do not extend to the other parts of the body.

Until the present, evaluation of DHS has consisted of only local MRI examination of spinal muscles. There are no reports on the association between skeletal muscle mass and sarcopenia.

Sarcopenia is defined as age-associated loss of skeletal muscle mass and function, and it includes a risk of adverse outcomes such as physical disability and poor quality of life [7, 8]. Sarcopenia is very common in older individuals, with a reported prevalence in 60- to 70-year-olds of 5–13% [9].

In a report on sarcopenia and spinal diseases, Miyakoshi et al. [12] reported 20% of Japanese patients with osteoporosis suffer complications due to sarcopenia, while only 10% of healthy individuals have sarcopenia. However, no studies have clearly defined the relationship between sarcopenia and DHS.

In the present study, a large proportion of DHS cases had sarcopenia compared to controls: 70 versus 25%. Muscle mass decrease was noted not only in the neck muscles but also throughout the entire body. Involvement of trunk and upper limb muscles in particular suggests a disuse atrophy of the upper body and spinal muscles. These results match those reported by Katz et al. for the shoulder blade and upper arm.

The pathogenesis of idiopathic DHS has not been elucidated. Regarding mechanisms of DHS, the spinal support muscles atrophy with age, resulting in sarcopenia. Furthermore, in cases of thoracolumbar kyphosis, due to decreases in spine flexibility, the load-bearing axis of the head shifts excessively forward, and mechanical load on the neck extensor muscle group is increased through the action of the lever arm. As a result, the extensor muscle group is continually extended, and a nonspecific inflammation occurs. Over time, changes in muscle quality take place; further irreversible decline of extensor muscle group strength is thought to occur, resulting in additional cervical spine kyphosis.

This study demonstrated that in the DHS, atrophy of upper limbs and upper truncal muscle become significant, suggesting that the maintenance of lean mass trunk and arm perhaps may prevent to lean due to a failure in functional cervical extensors. Further studies are needed to clarify the mechanism.

Our study has several limitations. (1) The first is that a small number of subjects were investigated, requiring confirmation of our findings in a larger population. (2) We did not conduct comprehensive measurements of whole spine alignment. (3) We did not evaluate muscles using MRI. (4) The study is a cross-sectional analysis, not a longitudinal one. (5) Dual-energy X-ray absorptiometry (DXA) seems to be the most reliable tool to evaluate body composition and is often considered the gold standard in clinical practice. BIA could provide a simpler, portative, and less expensive alternative. BIA has a tendency to overestimate muscle mass compared to DXA, but agreement between DXA and BIA is high for lean mass arm and for axial lean mass [13]. In the future, results should be compared to DXA and MRI measurements of muscle mass. (5) The appreciation of muscle mass has limits because BIA does not appreciate some qualities of muscle tissue such as fat infiltration and the muscle functionality. Moreover, this method underestimates the prevalence of sarcopenia in obese subjects and overestimates in lean subjects. (6) We evaluated only slim Japanese women with low BMI; therefore, the amount of truncal fat is much less likely to affect their calculations than in a typical western population. (7) Sometimes, there is a more rapid decline in muscle strength relative to muscle mass. However, we did not evaluate muscle strength.

## Conclusions

We examined the role of sarcopenia in DHS. We measured skeletal muscle mass in elderly women with idiopathic DHS using BIA. Sarcopenia was recognized in 70% of our DHS subjects, compared with 25% of controls. Total lean mass was decreased in the DHS group, especially lean mass trunk and lean mass arm, suggesting a disuse atrophy of the upper body and spinal support musculature. BIA can easily evaluate whole-body skeletal muscle mass. We expect it to contribute to further elucidating the pathogenesis of DHS in the future.

## Abbreviations

BIA: Bioelectrical impedance analyzer
DHS: Dropped head syndrome
DXA: Dual-energy X-ray absorptiometry
SMI: Skeletal muscle mass index
SVA: Sagittal vertical axis
